# A Case Report of Roundworms Causing Intestinal Obstruction in a Child

**DOI:** 10.1155/2024/6640941

**Published:** 2024-04-27

**Authors:** Tushar Krishnaraju, Suvendu Sekhar Jena, Amitabh Yadav, Samiran Nundy

**Affiliations:** Department of Surgical Gastroenterology and Liver Transplantation, Sir Ganga Ram Hospital, New Delhi, India

## Abstract

*Background*. Soil-transmitted helminth (STH) infections are a common global health issue that affects underprivileged communities without adequate access to clean drinking water, sanitation, and hygiene. *Ascaris lumbricoides* is the main species that infects humans. Among varied presentations, intestinal obstruction is common among children. Early detection of intestinal obstruction due to STH is critical to prevent severe complications. Here, we present the case of a 10-year-old child with acute intestinal obstruction due to roundworms. *Case Report*. A 10-year-old boy presented to the emergency department with recurrent abdominal pain, distention, and vomiting for three months with signs of peritonitis on examination. CT scan of the abdomen revealed roundworms in the distal ileum and a cocoon formed by adhesions of small bowel loops. Intraoperatively, worm balls were found in the proximal jejunum and ileum, causing small bowel obstruction, and a diseased segment of ileum had to be resected. The worms were removed, and an ileostomy was created. The patient was treated with albendazole and intravenous antibiotics such as ceftriaxone and metronidazole. An early closure of ileostomy was performed after 20 days. Histopathology showed roundworm eggs in the appendix and small bowel mucosa. *Discussion*. *Ascaris lumbricoides* infestation is a common cause of intestinal obstruction in children, and early detection is critical for avoiding severe complications. Prompt and appropriate treatment with antihelminthics and antibiotics is necessary to achieve a good outcome. In rare cases, surgical intervention may be required to treat intestinal obstruction caused by STH infections. In conclusion, the prevalence of STH infections highlights the need for public health interventions, such as improving access to clean water, sanitation, and hygiene, and for early detection and treatment to prevent severe complications such as intestinal obstruction.

## 1. Background

Evidence of infestation with *Ascaris lumbricoides* has been documented as far back as 1550 BCE in Ebers Papyrus from ancient Egypt. It was one of the three common parasitoses during the time of Hippocrates. *Ascaris* has been found in humans since the domestication of pigs. Soil-transmitted helminth (STH) infections are among the most common infections worldwide with an estimated 1.5 billion people or 24% of the world's population affected. These infections usually affect the low socioeconomic and underprivileged communities with inadequate access to clean drinking water, proper sanitation, and good hygiene in tropical and subtropical regions. The highest prevalence has been reported from sub-Saharan Africa, China, South America, and Asia [1]. Ascariasis is an important cause of intestinal obstruction in children presenting as an acute abdomen. An increased worm load may partially or completely obstruct the intestinal lumen. Most of the cases remain asymptomatic while symptoms like growth retardation, lower respiratory tract infections, hepatobiliary and pancreatic damage, and intestinal obstruction or peritonitis may be seen occasionally. Early detection of intestinal obstruction due to STH involves high suspicion and is essential for preventing serious and lethal complications [2]. Here, we present a case of a 10-year-old boy who presented to the emergency department with features of intestinal obstruction and was found to have ascariasis on evaluation.

## 2. Case Report

A 10-year-old boy presented with recurrent colicky pain in the lower abdomen for 3 months. Initially, the pain was mild, causing him to skip school occasionally, but relieved with nonnarcotic pain medication. He then developed more severe pain with associated abdominal distension, emesis, and complete absence of stool passage or flatus. He had completed antitubercular therapy one year back for Koch's abdomen (tuberculosis of the abdomen as informed by the mother, details of which were not known). There was no history of surgical interventions in the past. On examination, the abdomen was distended with tenderness, rebound tenderness in the lower abdomen, and localized guarding. A digital rectal examination showed an empty rectum with collapsed lumen.

**Figure 1:**
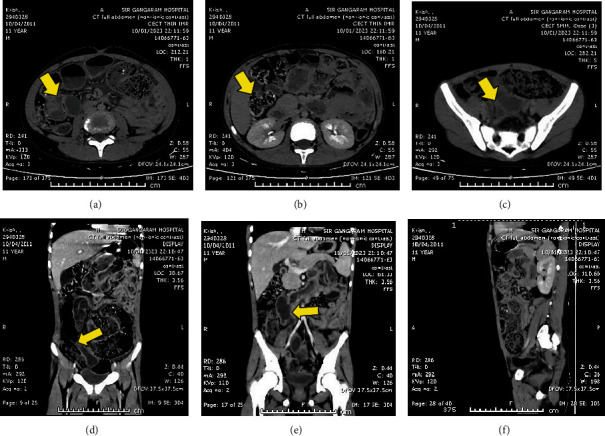
(a) NCCT abdomen shows dilated small bowel with air fluid levels. (b, c) Distal ileum and colon show feces with multiple foci of radiolucent, thread-like structures. (d) Coronal sections: dilated distal ileum filled with worms. (e) Clumping of small bowel into a cocoon, most likely due to Koch's abdomen in the past. (f) Sagittal section showing small bowel filled with feces and adult worms. Yellow arrows indicate important findings in each image.

He was evaluated with noncontrast CT imaging of the abdomen, which revealed thickening of the distal ileum with proximal small bowel dilation and subcentimeter mesenteric lymph nodes suggestive of possible tubercular involvement. Radiolucent thread-like foreign bodies in the terminal ileum also were concerning for possible intraluminal worms (Figure 1).

**Figure 2:**
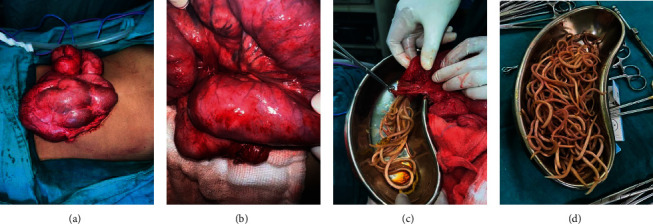
(a) Small bowel wrapped into a cocoon. (b) Roundworms in the small bowel lumen with hyperemic and edematous wall. (c, d) Extracted adult *Ascaris lumbricoides*.

He underwent exploratory laparotomy via a midline incision. Intraoperatively, there was 300 mL of ascitic fluid (culture was sterile with normal adenosine deaminase levels). Small bowel was encased in a cocoon and filled with roundworms. There was a formation of worm balls in the proximal jejunum and ileum causing obstruction. A segment of ileum, two feet away proximal to the ileocecal junction, was found to be ischemic and was resected. The worms were extracted both from proximal and distal bowel (Figure 2). An incidental appendectomy was performed, and a loop ileostomy was matured in the right iliac fossa.

**Figure 3:**
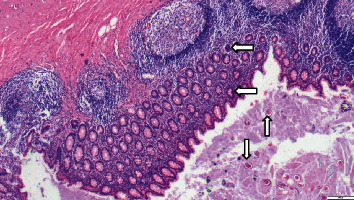
Section of the appendix on microscopy shows ova of *Ascaris* in the lumen and also embedded in the mucosal layer. Black arrows indicate ova.

Postoperatively, he was treated with syrup albendazole 400 mg, along with iv ceftriaxone and metronidazole. On POD 2, there was passage of a worm in the stoma. Foley's catheter was inserted in the distal loop of stoma and irrigated with albendazole solution for three consecutive days. Oral antihelminthics were changed to mebendazole for two weeks. The ileostomy was functioning well, and the child improved with treatment. After 20 days, abdominal CT with oral contrast and distal loopogram confirmed no residual worms in the small and large bowel. He underwent an early reversal of the ileostomy. He had an uneventful postoperative course and was discharged on postoperative day 6. Histopathology showed roundworm eggs in the appendix and small bowel mucosa and lumen with no evidence of tuberculosis. (Figure 3).

## 3. Discussion

Soil-transmitted helminth (STH) infections are among the most common infections worldwide with an estimated 1.5 billion people or 24% of the world's population affected. *Ascaris lumbricoides* is the main species that infect humans. Risk factors include low socioeconomic status and poor access to clean water, sanitation, and hygiene [1–3].

**Figure 4:**
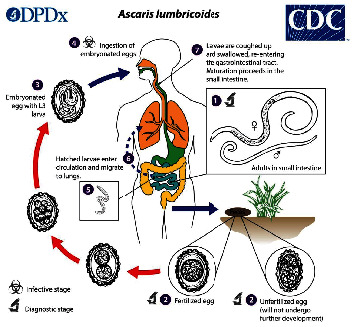
Life cycle of roundworm. Source: Centre for Disease Control and Prevention [5].

*Ascaris lumbricoides* enters the human host by ingestion of the fertilized eggs that contaminate the soil in poorly sanitized areas. Hatched larvae then enter the circulation and migrate to the lungs where they are then coughed up and swallowed and reenter the gastrointestinal tract. The worms mature in the small intestine. The life cycle is perpetuated by fecal excretion of eggs back into the soil (Figure 4). The eggs passed in feces will take about 3 weeks to mature in the soil before they become infective. Hence, there is no direct transmission from person to person or infection from fresh feces. Roundworms cannot multiply in the human host. Therefore, reinfection can only occur as a result of contact with infective stages in the environment [1, 3, 4].

Many people with mild roundworm infestations are asymptomatic as they remain in the lumen of the gut without releasing toxins. However, due to the high prevalence of the disease, the global burden of symptomatic disease remains relatively high. The development of symptomatic disease is proportional to the high worm load in individuals [2]. Roundworms causing intestinal obstruction are more common in children because of the smaller diameter of the lumen of the bowel and increased worm load [4].

Symptomatic cases are now quite rare, and a high index of suspicion is necessary to make a prompt diagnosis. “Massive ascariasis” is a term used for overwhelming infestation by roundworms [3]. Symptoms include colicky abdominal pain, vomiting, and constipation. Possible complications may include worms in the vomitus, ileocecal intussusception, volvulus, gangrene, and perforation of the bowel [6]. Neurotoxins are excreted by roundworms which can cause contraction of the small bowel (spasticity), resulting in obstruction. An inflammatory reaction in bowel segments may be caused due to the other toxins excreted by the worms, such as anaphylatoxins, hemolysins, and endocrinolysins [6].

In children with heavy worm infestation, large aggregates of worms can be seen as “cigar bundles,” which are radiolucent areas on plain radiograph of the abdomen. Occasionally, the contrast between a mass of worms and the gas in the bowel produces a “whirlpool” effect. Ultrasonography of the abdomen is fast, safe, noninvasive, and a low-cost modality for suspected intestinal worm infestation, and various appearances of roundworms have been described like a thick echogenic strip with a central anechoic tube or multiple long, linear, parallel echogenic strips without acoustic shadowing. On computed tomography imaging, worms can be visualized on soft tissue window as multiple, linear thread-like structures [4].

The medical management is constant, with oral albendazole 400 mg as a single dose as the drug of choice. The second choice of treatment is mebendazole 100 mg twice a day for three days or 500 mg as a single dose or ivermectin 100 microgram/kg to 200 microgram/kg once. Older treatments like Racine oil (15 to 30 mL) administered through the Ryle's tube (nasogastric tube), followed by piperazine (75 mg/kg), are no longer followed. The hypertonic saline enema, which causes irritation and promotes disentangling and expulsion of colonic worms, is rarely used. The usage of antihelminthic agents as a part of conservative management in patients with intestinal obstruction is debatable. They may cause serious complications such as intussusceptions, volvulus, hemorrhage, gangrene, or even perforation as they alter the motility of the worms and hamper their clearance [4].

However, the decision for surgery varies. Features of acute obstruction and toxic-looking children (dehydration with tachypnoea, tachycardia, sweating, raised temperature, decreased urine output, etc.) require urgent surgical intervention. Enterotomy and extraction of worms were discontinued due to complications such as burrowing of adult worms through the sutures and spillage into the peritoneal cavity. Milking of worms into the colon may be sufficient in obstruction with viable bowel. In case of intestinal compromise, resection and anastomosis with or without diverting proximal stoma can be performed. Anastomosis without diversion may be dangerous in cases of high worm load as the worms are known to burrow through the site of anastomosis and cause peritonitis and anastomotic leak [6].

In 2001, delegates to the World Health Assembly unanimously approved a resolution (item WHA 54.19), which urged endemic countries to start aggressively combating worms, especially schistosomiasis and soil-transmitted helminths. World Health Organization (WHO) advises all at-risk individuals living in endemic areas to undergo periodic medical deworming without first receiving a personal diagnosis. In endemic nations in 2021, more than 500 million children received antihelminthic treatments, or 62% of all children at risk [1].

Six global targets have been set by the WHO for soil-transmitted helminthiases by the year 2030, including the elimination of STH morbidity in children of preschool and school age, reducing the number of tablets, increasing domestic financial support, establishing an effective strongyloidiasis control programme for school-age children, establishing an effective programme to control STH in pregnant and lactating women, and ensuring that everyone has access to at least the most basic sanitation and hygiene by the year 2030 in STH-endemic areas which are all goals [1].

According to estimates, 225 million children in India are at risk for STH. Numerous health initiatives have been carried out, such as mass drug administration, the WASH intervention (water, sanitation, and hygiene), the “Total Sanitation Campaign,” and the “Swacch Bharat Abhiyan,” which was launched by the Indian government to install flush pit latrines and community mobilization among villagers [7].

## 4. Conclusion

Intestinal ascariasis arises from risk factors such as low socioeconomic conditions, poor nutrition, and lack of awareness. Health education is of utmost importance in preventing and/or controlling the disease. Medical treatment with antihelminthics is the mainstay of treatment. However, the dosing should be adjusted accordingly. Surgery is indicated in emergency situations such as intestinal obstruction or perforation of the bowel. In case of hyperemic or unhealthy bowel on operation, a diverting stoma is a valuable option. Bowel irrigation with albendazole solution may be more effective and less detrimental than hypertonic saline. Due to high rates of reinfection, periodic administration of antihelminthics every three months for the next three cycles was carried out.

## Abbreviations

NCCT: Noncontrast computed tomography
STH: Soil-transmitted helminth
WHO: World Health Organization.

## References

[1] Soil-transmitted helminth infections. https://www.who.int/news-room/fact-sheets/detail/soil-transmitted-helminth-infections.

[2] Elmi A. M., Çelik C., Jama S. M., Dirie A. M. (2022). Intestinal obstruction in a child with massive ascariasis and associated acute appendicitis: a case report. *Annals of Medicine and Surgery*.

[3] Turyasiima M., Matovu P., Kiconco G. (2021). Intestinal obstruction in a child with massive ascariasis. *Case Reports in Pediatrics*.

[4] Mishra P. K., Agrawal A., Joshi M., Sanghvi B., Shah H., Parelkar S. V. (2008). Intestinal obstruction in children due to ascariasis: a tertiary health centre experience. *African Journal of Paediatric Surgery*.

[5] CDC-Centers for Disease Control, Prevention. https://www.cdc.gov/parasites/ascariasis/biology.html.

[6] Villamizar E., Mendez M., Bonilla E., Varon H., de Ontra S. (1996). Ascaris lumbricoides infestation as a cause of intestinal obstruction in children: experience with 87 cases. *Journal of Pediatric Surgery*.

[7] Abraham D., Kaliappan S. P., Walson J. L., Rao Ajjampur S. S. (2018). Intervention strategies to reduce the burden of soil-transmitted helminths in India. *Indian Journal of Medical Research*.

